# Comprehensive Optimization of a Freeze-Drying Process Achieving Enhanced Long-Term Stability and In Vivo Performance of Lyophilized mRNA-LNPs

**DOI:** 10.3390/ijms251910603

**Published:** 2024-10-01

**Authors:** Teresa Alejo, Alfonso Toro-Córdova, Laura Fernández, Andrea Rivero, Andrei Mihai Stoian, Luna Pérez, Victor Navarro, Juan Martínez-Oliván, Diego de Miguel

**Affiliations:** CerTest Biotec S.L., 50840 San Mateo de Gállego, Spain; talejo@certest.es (T.A.); atoro@certest.es (A.T.-C.); lfernandez@certest.es (L.F.); arivero@certest.es (A.R.); amihai@certest.es (A.M.S.); lperez@certest.es (L.P.); vnavarro@certest.es (V.N.)

**Keywords:** mRNA-LNPs, freeze-drying/lyophilization, thermostable vaccines

## Abstract

The success of mRNA vaccines against SARS-CoV-2 has prompted interest in mRNA-based pharmaceuticals due to their rapid production, adaptability, and safety. Despite these advantages, the inherent instability of mRNA and its rapid degradation in vivo underscores the need for an encapsulation system for the administration and delivery of RNA-based therapeutics. Lipid nanoparticles (LNPs) have proven the most robust and safest option for in vivo applications. However, the mid- to long-term storage of mRNA-LNPs still requires sub-zero temperatures along the entire chain of supply, highlighting the need to develop alternatives to improve mRNA vaccine stability under non-freezing conditions to facilitate logistics and distribution. Lyophilization presents itself as an effective alternative to prolong the shelf life of mRNA vaccines under refrigeration conditions, although a complex optimization of the process parameters is needed to maintain the integrity of the mRNA-LNPs. Recent studies have demonstrated the feasibility of freeze-drying LNPs, showing that lyophilized mRNA-LNPs retain activity and stability. However, long-term functional data remain limited. Herein, we focus on obtaining an optimized lyophilizable mRNA-LNP formulation through the careful selection of an optimal buffer and cryoprotectant and by tuning freeze-drying parameters. The results demonstrate that our optimized lyophilization process maintains LNP characteristics and functionality for over a year at refrigerated temperatures, offering a viable solution to the logistical hurdles of mRNA vaccine distribution.

## 1. Introduction

Following the tremendous success of mRNA vaccines in protecting against the disease caused by SARS-CoV-2 infection, mRNA-based drugs are being widely studied as candidates for various pharmaceutical products [1]. Compared to conventional vaccines, which are relatively laborious and slow to produce, mRNA vaccines can be designed and scaled up more quickly and are versatile, easily adaptable, highly effective, and non-toxic since they do not integrate or alter genomic DNA [2]. However, RNA is a molecule with high chemical instability and is rapidly degraded by ribonucleases in the extracellular environment, preventing its entry into target cells. This poses an important limitation for using RNA as a free molecule and underscores the need for an encapsulation system for the administration and delivery of RNA-based therapeutics. Among the encapsulation systems that have been studied, two stand out for their demonstrated effectiveness and robustness: lipoplexes and lipid nanoparticles (LNPs). Both systems are based on similar lipid mixtures, usually comprising an ionizable/cationic lipid, a helper lipid, and cholesterol. However, they differ in size, heterogeneity, and the location of the mRNA molecules they transport: while in lipoplexes the mRNA is adsorbed on their surface, in LNPs it is encapsulated inside. Although both systems work well in vitro, LNPs have proven to be more robust, safe, and effective for in vivo use [3].

Another important aspect of mRNA therapeutics are the strict requirements to preserve mRNA integrity during manufacturing, storage, and transportation, which directly affects the logistics chain and the final product price [4]. While conventional vaccines based on inactivated microorganisms can be stored under refrigeration conditions (2–8 °C) for at least 6 months, currently approved mRNA vaccines require ultra-low temperatures for storage, hampering their distribution, especially in developing countries with structural limitations. In this regard, numerous studies have been conducted aiming at finding optimal freezing conditions for LNPs to minimize chemical damage [4]. To that end, cryoprotectants such as sucrose are added to mRNA-LNP formulations to preserve structural and functional properties during the freezing process. Thus, both mRNA-based vaccines authorized against COVID-19, SpikeVax^®^ and Comirnaty^®^, contain a 10% sucrose content in the final product (prior to dilution). Notably, although sucrose is used as a cryoprotective agent in both cases, the initial storage temperature requirements were different, as SpikeVax^®^ required storage between −15 °C and −20 °C while Comirnaty^®^/BNT162b2 necessitated storage between −60 °C and −80 °C [5]. In 2021, the Comirnaty^®^ formulation was updated by replacing the originally used PBS (phosphate-buffered saline) with Tris (abbreviation of tris(hydroxymethyl)aminomethane) as the buffering agent, which improved its stability and allowed the approval by the European Medicines Agency (EMA) for storing the BioNTech/Pfizer vaccine between −15 °C and −25 °C for up to two weeks [6]. Nevertheless, the mid- to long-term storage of mRNA-LNPs vaccines still requires infrastructure capabilities to ensure sub-zero temperatures along the entire chain of supply. While these requirements have been addressed from an engineering perspective by using special shipping containers and ultra-freezers, this still poses a logistical challenge that limits distribution and significantly increases the final cost, highlighting the need to develop biotechnological advancements to improve mRNA vaccine stability under non-freezing conditions to facilitate logistics and distribution [4].

Lyophilization presents itself an effective alternative to prolong the shelf life of mRNA vaccines under refrigeration conditions, without the need for sub-zero temperatures. Lyophilization is a relatively mild drying method used in the pharmaceutical industry to improve the stability of biomolecules and drugs, based on removing water molecules from formulations through sublimation under a high vacuum and low temperature [7]. There is currently an enormous interest in obtaining lyophilized formulations of mRNA-LNPs. However, the lyophilization of mRNA-LNPs is a complex process since freezing and dehydration impose mechanical stress and deformation on lipid structures, leading to LNP aggregation and the release of encapsulated mRNA [8,9]. In this regard, the physicochemical parameters of LNPs such as particle size, polydispersity, and encapsulation efficiency are critical for their bioactivity and efficacy and therefore must be preserved during the lyophilization process and subsequent storage. During the last few years, several studies have described lyophilized mRNA-LNPs maintaining their activity compared to liquid formulations [10,11,12,13]. However, only two studies show information about the long-term stability of lyophilized mRNA-LNPs [11,12]. Regarding commercial products, the GEMCOVAC-19 vaccine authorized for use in India consists of a lyophilized formulation based on mRNA encoding the SARS-CoV-2 S protein. This vaccine is composed of a lipoplex consisting of DOTAB and squalene, stabilized with polysorbates, and uses sucrose as a cryoprotectant; however, it does not use LNPs as a vehicle. According to the authors, this product can be stored under refrigeration and preserves its activity for 21 months at room temperature, although details on stability profile are not publicly available [4].

The evidence obtained so far underscores that, to obtain robust lyophilizable mRNA-LNPs formulations, it is imperative to comprehensively explore the various factors involved in the lyophilization process, such as the choice of buffers and cryoprotectants or the addition of excipients, as well as the intrinsic parameters of the lyophilization process, such as the temperature and pressure used. Thus, in the present study, we describe the systematic optimization process of a lyophilizable mRNA-LNP formulation, first paying attention to the type of buffer and lyoprotectant used and then adjusting the physical variables involved in the different stages of the freeze-drying process until obtaining the optimal conditions required to maintain the physicochemical and functional attributes of LNPs. Moreover, a long-term stability study was carried out for over one year, demonstrating that the lyophilization process managed to maintain the characteristics and in vivo functionality of LNPs under a refrigerated temperature throughout the studied period, without the need for freezing. Altogether, these results validate our freeze-drying process and pave the way for the systematic application of this methodology to obtain lyophilized mRNA-LNPs.

## 2. Results and Discussions

### 2.1. Effect of Buffers and Lyoprotectants in the Freeze-Drying of LNPs

**Figure 1:**
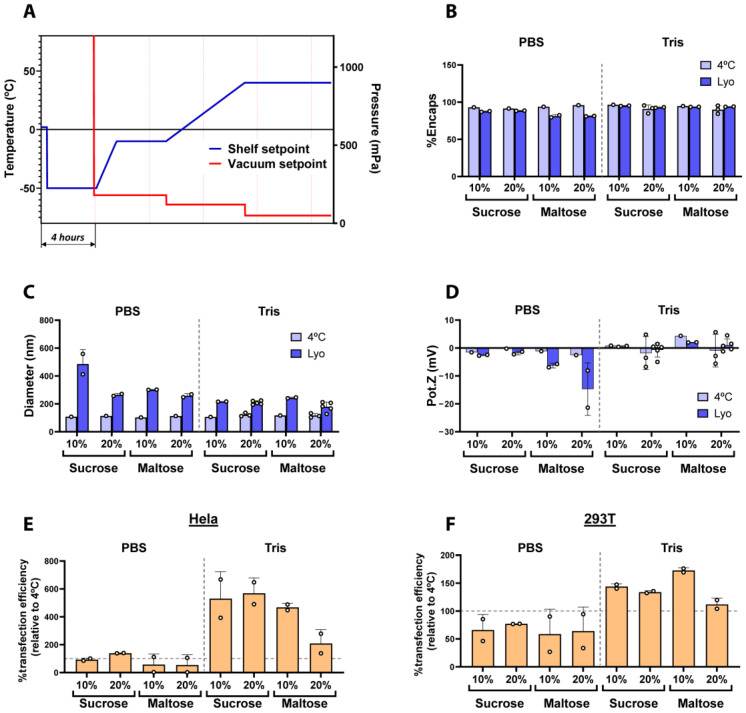
(**A**) Shelf temperature and vacuum setpoints used during the freeze-drying cycle. (**B**–**D**) Comparison of the physicochemical parameters of fresh (4 °C) and freeze-dried LNPs in two different buffers (PBS or Tris 5 mM) using sucrose or maltose as lyoprotectants. (**B**) Encapsulation efficiency of mRNA (%). (**C**) Particle size obtained by DLS and (**D**) the Z potential values. Dashed line indicates separation of the PBS and Tris groups. (**E**,**F**) Transfection efficiency of freeze-dried LNPs normalized with control LNPs in HeLa and 293T (**F**) cells. Vertical dashed line indicates separation of the PBS and Tris groups, horizontal dashed lines indicate the basal transfection efficiency respective to control non-lyophilized LNPs. Data are presented as the geometric mean of at least three independent replicates, and error bars indicate the standard deviation (±SD).

First, we sought to define the optimal conditions to preserve lyophilized LNPs. To that end, we evaluated the effect of different buffers and lyoprotectants on the physicochemical parameters and activity of freeze-dried LNPs. As a starting point, we used a standard freeze-drying recipe based on our previous experience with other products, which comprised the following steps: a freezing step at −50 °C, followed by a primary drying step at −10 °C and 180 mTorr, and finally a secondary drying step at 40 °C using 120 mTorr and 50 mTorr (Figure 1A). Table S1 summarizes the lyophilization protocol used to obtain the LNPs used in Figure 1. Notably, all LNPs used in this study were prepared following a standard LNP formulation and using commercially available and clinically relevant components. More specifically, the LNPs were composed of SM-102 as an ionizable lipid, DOPE (Dioleoylphosphatidylethanolamine) as a helper lipid, cholesterol, and DMG-PEG (1,2-Dimyristoyl-rac-glycero-3-methoxypolyethylene glycol-2000) at molar ratios of 50:10:38.5:1.5, respectively. Of note, these lipid molar ratios correspond to the Onpattro^®^ (Patisiran) LNP formulation, currently considered in the field as the standard lipid ratio for LNP formulations [14,15] and used in the Spikevax^®^ SARS-CoV-2 vaccine [5]. Notably, in our case, we included DOPE as a helper lipid instead of DSPC (1,2-dioctadecanoyl-sn-glycero-3-phosphocholine) due to its ability to enhance endosomal scape through the formation of membrane hexagonal H II phase structures [16]. The percentage of encapsulated mRNA, hydrodynamic diameter, zeta potential, and in vitro transfection in HeLa and 293T cells were analyzed in both non-lyophilized and reconstituted LNPs (Figure 1).

As illustrated in Figure 1B–F, we evaluated the impact of two different buffers routinely used in the preparation of mRNA-encapsulated LNPs, PBS and 5 mM Tris (both at pH 7.4), as well as the use of sucrose and maltose as lyoprotectants at either 10 or 20 *w*/*v*% on the physicochemical properties of freshly prepared and freeze-dried LNPs. As shown in Figure 1B, in general, the encapsulation efficiency of LNPs was preserved when 5 mM Tris buffer was used for the lyophilization of LNPs in comparison to fresh LNPs, regardless of the lyoprotectant used, whereas LNPs prepared in PBS showed a decrease in mRNA encapsulation after lyophilization, being less than 80% with maltose as the lyoprotectant. On the other hand, and in line with previous reports on freeze-dried LNPs [11,12,13], we observed a general increase in particle diameter after lyophilization (Figure 1C). Nevertheless, the size increment was especially noticeable in those LNPs buffered with PBS, whilst the use of Tris generally maintained lower diameter values after the freeze-drying process, regardless of the lyoprotectants.

Interestingly, the zeta potential of LNPs prepared in PBS rendered more negative values than those prepared in Tris (Figure 1D). This effect could, at least partially, be attributed to the leakage of negatively charged mRNA molecules from the LNPs [17], given the correlation between the most negative Z potential results and the higher drop in mRNA encapsulation observed in Figure 1B. However, the zeta potential did not significantly change after the lyophilization of LNPs prepared in Tris 5 mM, pointing to the superior stability of the formulations in this buffer. Recent studies [18] demonstrated that Tris yielded better cryoprotection and transfection efficiency for LNPs stored frozen at −20 °C compared to PBS. The improved stability of LNPs in Tris buffer can be attributed to the avoidance of oxidation processes by the reaction of Tris with aldehyde impurities, thereby reducing the formation of mRNA–lipid adducts [19]. Moreover, it is believed that PBS buffers do not correctly preserve the pH of solutions after freezing them in the presence of sodium ions, contributing to the LNPs’ instability [20]. These results are in agreement with ours, where Tris preserved the LNPs’ properties better compared to PBS. Finally, all LNPs were transfected in Hela and 293T cells to test their transfection ability. As observed in Figure 1E and 1F, LNPs prepared in Tris buffer led to higher transfection efficiencies in both cells lines, regardless of the lyoprotectant used, whereas LNPs formulated in PBS rendered generally lower transfection results. This result is in line with the leakage of RNA and the change in their nanoparticle size and Z potential that was observed when PBS was used. Moreover, Figure S1 shows marked differences in cake appearance depending on the use of PBS or Tris buffers, leading to collapsed cakes in the case of PBS and well-formed homogeneous cakes when Tris was used as the buffer.

Altogether, our results demonstrate that Tris is more efficient than PBS at preserving the physico-chemical and functional properties of mRNA-LNPs during freeze-drying, independently of the lyoprotectants used. Based thereupon, we selected Tris buffer for the upcoming experiments.

### 2.2. Optimization of the Freeze-Drying Process Parameters

Next, we focused on improving the physicochemical properties and biological activity of LNPs by modifying different critical parameters known to influence the lyophilization process. In addition, and in light of the small differences obtained in the first study when comparing sucrose vs. maltose as lyoprotectants at different concentrations, we used a fixed concentration of 20% (*w*/*v*).

**Figure 2:**
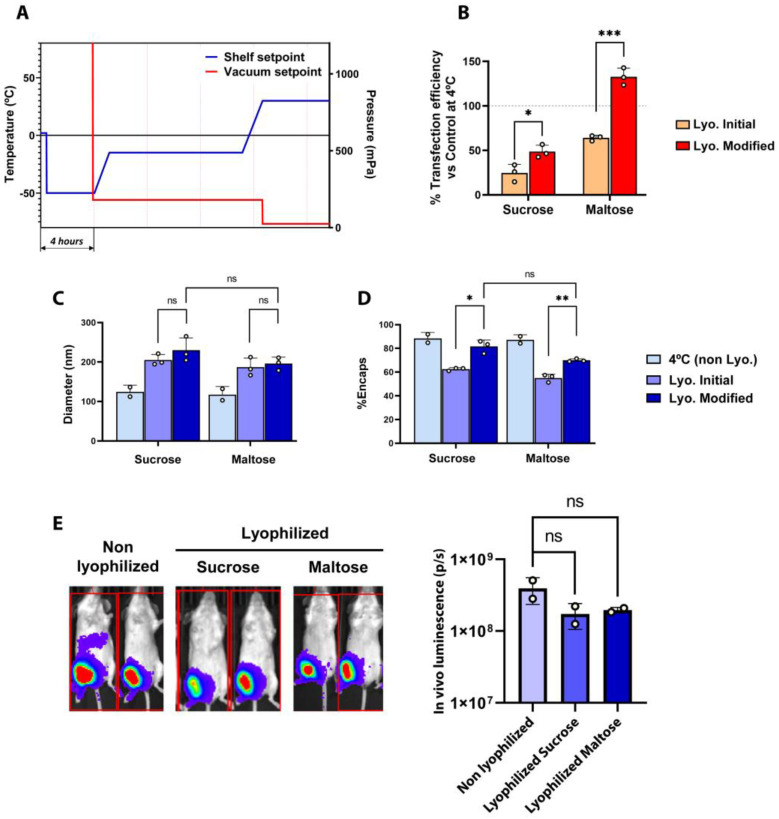
(**A**) Shelf temperature and vacuum setpoints used during optimized freeze-drying. (**B**) Transfection efficiency of freeze-dried LNPs normalized with control samples at 4 °C in 293T cells. Horizontal dashed lines indicate the basal transfection efficiency respective to control non-lyophilized LNPs. (**C**,**D**) Comparison of the physicochemical parameters of fresh (4 °C) and freeze-dried LNPs obtained by the initial and modified methods, in 20% sucrose or 20% maltose. (**B**) Particle size obtained by DLS. (**C**) Encapsulation efficiency of mRNA (%). (**E**) Average luminescence radiance (p/s) and bioluminescence images of mice treated with mRNA-LNPs. Mice were intramuscularly injected with LNPs at a dose of 1 µg of LUC-encoding mRNA/animal, and bioluminescence images were taken four hours post inoculation using the IVIS Lumina XRMS Imaging System. For graphs (**B**–**D**), data are presented as the geometric mean of at least three independent replicates, and error bars indicate the standard deviation (±SD). For the graphs in (**E**), data are the geometric mean of independent duplicates (two mice injected per sample), and error bars indicate the standard deviation (±SD). * *p* < 0.05, ** *p* < 0.01, *** *p*< 0.005, ns = not significant.

To improve the lyophilization process, the first step was to perform a comprehensive evaluation of the evolution of the temperature and vacuum values obtained with the initial lyophilization recipe used in Figure 1. As observed in Figure S2, in the initial lyophilization recipe, the differences between the Pirani gauge and the capacitance manometer revealed that there was still a considerably amount of remnant humidity at the end of the primary drying step, implying that the primary drying process had not been completed before proceeding towards the secondary drying step. Additionally, we also observed that the product probe temperature did not reach the stablished set point temperature (−10 °C) at the end of the primary drying step. Considering these results, the length of the primary drying step was extended to ensure that free water from ice crystals was completely removed before proceeding towards secondary drying, where only the interstitial water molecules are removed [21,22]. To avoid damage to the LNPs during primary drying and aiming at stablishing less aggressive conditions that would help preserve the structure of LNPs throughout the lyophilization process, the temperatures of primary and secondary drying were set to −15 °C and 30 °C, respectively. The parameters used in the optimized lyophilization protocol are summarized in Table S2. A comparison of fresh and lyophilized LNPs using the initial and the optimized protocol is illustrated in Figure 2. Functionally, freeze-dried LNPs prepared with the optimized method presented an overall higher transfection potential than those obtained with the initial process, especially when maltose was used as the lyoprotectant (Figure 2B). On the other hand, particle size was increased in all cases upon lyophilization, regardless of the protocol or the lyoprotectants used, although the use of maltose somehow showed more consistent results for both the initial and optimized protocols (Figure 2C). Regarding mRNA encapsulation, LNPs lyophilized using the optimized method presented better results in comparison to the initial protocol, greatly reducing the loss of mRNA encapsulation during the freeze-drying process (Figure 2D). Analyzing the process parameters, and differently from the results obtained with the initial freeze-drying recipe, the values of both the Pirani gauge and the capacitance manometer converged at the end of the primary drying step, indicating the nearly complete removal of water molecules before starting secondary drying (Figure S3).

Finally, once we established the impact of the different variables on the physico-chemical properties of LNPs, we assessed the in vivo performance of LNPs lyophilized with either sucrose or maltose as compared to fresh, non-lyophilized LNPs. As depicted in Figure 2E, lyophilized LNPs showed remarkable in vivo activity compared with their fresh, non-lyophilized counterparts, achieving a comparable luciferase expression upon the intramuscular administration of 1 µg of mRNA-LNPs in mice, demonstrating that the freeze-drying process did not reduce the mRNA-LNPs’ functionality. Notably, both sucrose and maltose at 20% showed pretty similar results in both Figure 1 and Figure 2, with the type of buffer being the most relevant variable. Nevertheless, some differences could be observed in the functional results showed in Figure 1E, F and in Figure 2B. Ideally, lyophilized LNPs would functionally behave as similarly as possible to their non-lyophilized counterparts. Applying this criterium, 20% maltose presents the most consistent results. This, together with the slightly lower variability observed in vivo (Figure 2E), led us to select 20% maltose as our preferred lyoprotectant.

Altogether, the results demonstrate that the modifications that were introduced with respect to the initial lyophilization recipe successfully achieved an improvement in the process, which was in turn reflected in the form of better and more efficient lyophilized LNPs.

### 2.3. Assessment of Long-Term Stability of Freeze-Dried Lipid Nanoparticles

Once we confirmed the improved performance and in vivo functionality of freeze-dried LNPs obtained with the optimized lyophilization recipe at day 0 post lyophilization, we set out to assess the long-term stability of these LNPs. To that end, we designed a 1-year stability experiment, comparing LNPs lyophilized using the optimized recipe and the best conditions found in previous experiments (Tris 5 mM supplemented with 20 *w*/*v*% maltose) and stored at 4 °C, 25 °C, and 37 °C, with non-lyophilized LNP formulations kept at the standard conditions used for the long-term storage of mRNA-LNPs [23]: frozen at −80 °C and kept in Tris 5 mM supplemented with 15% (*w*/*v*) sucrose as cryoprotectant. All the different experimental conditions were prepared with LNPs from the same production batch to reduce experimental variability.

As shown in Figure S4A, the dried LNPs resulted in a white cake with a non-collapsed and homogeneous aspect, which rapidly dissolved upon reconstitution with water. Figure S4 shows the physicochemical characterization of freshly prepared and freeze-dried LNPs prepared using the optimized method. As expected, and in line with previous experiments, the lyophilization process caused certain variations in particle size, Z potential, and RNA encapsulation, but in all cases, they did not greatly differ from freshly prepared LNPs (Figure S4B–E).

**Figure 3:**
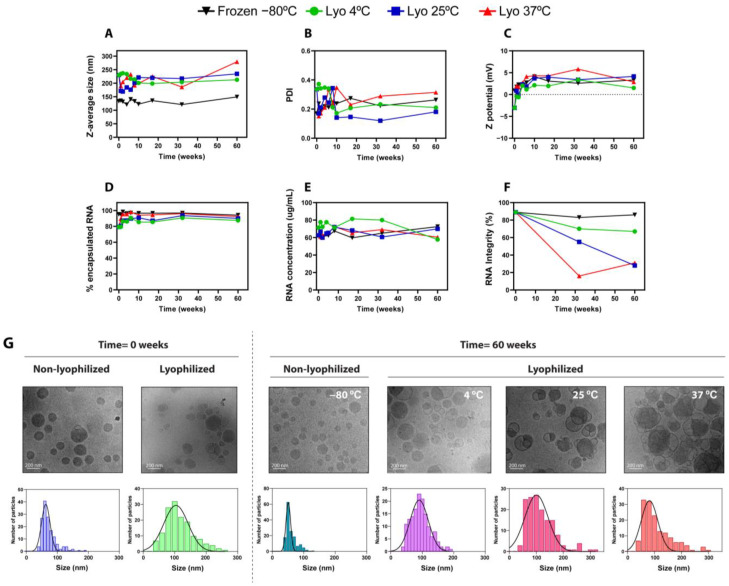
Physicochemical characterization of frozen (−80 °C) and freeze-dried LNPs stored at 4 °C, 25 °C, and 37 °C for up to 60 weeks. (**A**–**F**) Analysis of physicochemical properties: (**A**) particle size, (**B**) polydispersity index, (**C**) Z potential, (**D**) encapsulation efficiency (%) of mRNA, (**E**) total mRNA concentration obtained by RiboGreen assay, and (**F**) mRNA integrity (%) obtained by capillary electrophoresis. (**G**). Cryo-TEM images of liquid and lyophilized LNPs freshly prepared or stored at 4 °C, 25 °C, and 37 °C for 60 weeks. For each image, size distribution histograms with Gaussian fitting curves (solid lines) are depicted, obtained from the Cryo-TEM images (N = 150/image).

**Figure 4:**
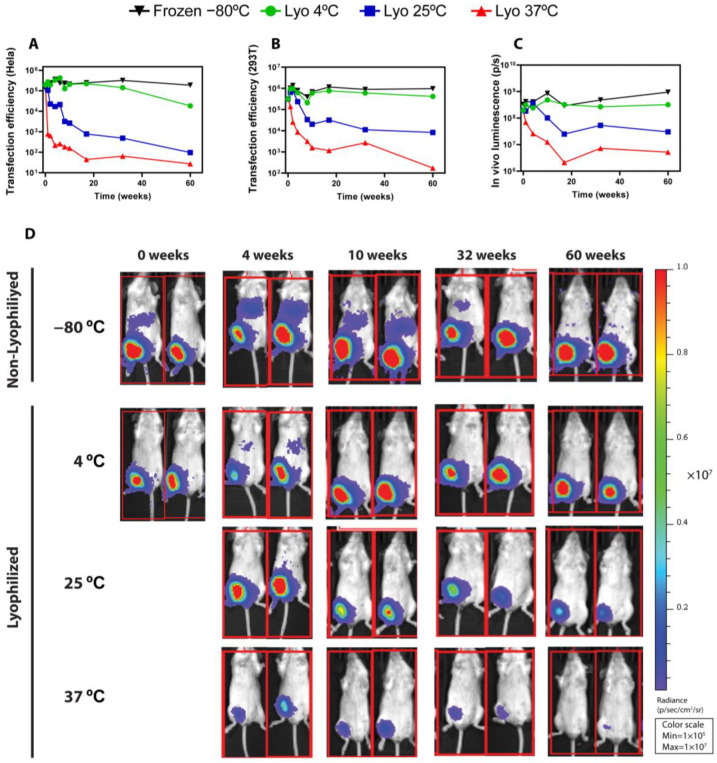
Functional results of frozen (−80 °C) and freeze-dried LNPs stored at 4 °C, 25 °C, and 37 °C for up to 60 weeks. (**A**,**B**) Transfection efficiency of the indicated LNPs in HeLa (**A**) and 293T (**B**) cells. (**C**,**D**) Average luminescence radiance (**C**) and bioluminescence images (**D**) of mice treated with mRNA-LNPs. Mice were intramuscularly injected with LNPs at a dose of 1 µg of LUC-encoding mRNA/animal, and bioluminescence images were taken four hours post inoculation using the IVIS Lumina XRMS Imaging System (software version 4.8.2).

Next, once we assessed the initial physico-chemical characteristics, we launched a long-term stability study with different experimental conditions for up to 12 months. As shown in Figure 3, the size (Figure 3A) and PDI (Figure 3B) of frozen (−80 °C) and freeze-dried LNPs (stored at 4 °C, 25 °C, and 37 °C) did not significantly change over 12 months. This behavior indicates that LNPs remained stable and homogeneous during the analyzed period, and it is in line with previous observations of benchmark LNPs kept at different storage conditions [24]. Z potential values (Figure 3C) slightly increased over time during the first weeks, both in frozen and lyophilized formulations, probably due to a small shift in the pH of the solutions [4,24] that could slightly modify the ionization status of the amine groups. In this regard, other authors already described similar deviations in the Z potential during the first days of storage [12]. It is important to note that these small initial variations did not affect other physical parameters such as encapsulation efficiency or particle size nor the functionality of LNPs (Figure 4). Intriguingly, the encapsulation efficiency of lyophilized LNPs appeared to increase around 10% during the first weeks (Figure 3D), a phenomenon that has already been reported in other stability studies of lyophilized LNPs [17], and that can be attributed to the degradation of non-encapsulated mRNA at the storage conditions while encapsulated mRNA is protected from thermo-degradation. In accordance with this, the concentration of encapsulated mRNA (Figure 3E) did not show significant changes over time in all conditions tested. Interestingly, despite the mRNA being successfully encapsulated and retained within the LNPs in all cases, the mRNA integrity of lyophilized LNPs stored at 25 °C and 37 °C dropped significantly after 1 year of storage in accelerated conditions (Figure 3F). Nonetheless, lyophilized LNPs stored at room temperature (25 °C) retained above 50% mRNA integrity for 32 weeks, preserving functional mRNA within the LNPs even under non-cold conditions. Remarkably, the integrity of mRNA encapsulated within lyophilized LNPs stored during 1 year at 4 °C was comparable to that from LNPs kept at −80 °C.

Next, liquid and lyophilized LNPs were characterized using Cryo-TEM to evaluate particle morphology and structure. Figure 3G depicts Cryo-TEM images of liquid and lyophilized LNPs when they were freshly prepared and after storage for 60 weeks. In line with the typical LNP structure described in the literature [25], freshly prepared, non-lyophilized LNPs displayed a spherical shape with uniform size and a typical solid-core morphology. Intriguingly, certain bleb structures were observed in lyophilized LNPs, characterized by the presence of an electron-dense core and an aqueous compartment surrounded by a lipid bilayer. Bleb structures in LNPs have been previously reported [26], and they are described to enhance transfection efficiency both in vitro and in vivo, improving intracellular delivery and endosomal scape [27]. Our results demonstrate that the in vivo efficacy is preserved after lyophilization (Figure 4), so the presence of blebs does not negatively impact mRNA-LNP functionality. LNPs stored at 4 °C for 60 weeks in both liquid and lyophilized forms preserved their structure; however, the freeze-dried LNPs stored at 25 °C and mainly at 37 °C became more structurally heterogeneous. These morphological alterations, together with the observed drop in the mRNA integrity of lyophilized LNPs after 1 year storage at 25 °C and 37 °C, may justify the decrease in activity under accelerated storage conditions. Finally, size distribution histograms and Gaussian fitting curves were also stablished from the Cryo-TEM images, corroborating the average size and distribution determined using DLS.

As briefly outlined above, the functional results of lyophilized LNPs, both in vitro and in vivo, correlate with the physico-chemical results obtained for the different conditions. As shown in Figure 4A,B, the transfection efficiency in Hela and 293T cells was seamlessly maintained over the entire period of time for both lyophilized LNPs stored at 4 °C and frozen (−80 °C) LNPs. On the other hand, the functionality of LNPs kept at 37 °C decreased drastically over time, whereas lyophilized LNPs kept at room temperature retained certain transfection activity. Remarkably, these results were further confirmed in vivo, where the average luminescence radiance (Figure 4C) was preserved during 1 year for both lyophilized LNPs stored at 4 °C and frozen (−80 °C) LNPs. It should be noted that, in lyophilized LNPs stored at room temperature (25 °C), the bioactivity remained unaffected for at least 4 weeks. From that point onwards, the bioluminescence decreased gradually over time, although in vivo functionality was still robustly observed after 32 weeks and could still be detected after 60 weeks, as shown in the time course of the luminescence images (Figure 3C,D). In accelerated conditions (37 °C), the activity was decreased from the first week, and at the end of the study, only residual protein expression could be detected in vivo. Non-lyophilized LNPs stored under frozen conditions (−20 °C and −80 °C) (Figure S5I,J) retained most of the transfection potential, whereas non-lyophilized LNPs stored at 4 °C (Figure S5I,J) showed a stark activity decrease after 4 weeks. Importantly, this contrasts with lyophilized LNPs at 4 °C, whose in vivo expression remained largely unaffected for 1 year.

In brief, our optimized freeze-drying process was able to produce lyophilized LNPs which retained their in vivo bioactivity at an almost unaffected level for 1 year when stored at 4 °C. This long-term storage at temperatures achievable by regular fridges could overcome cold chain logistic limitations and could be crucial for facilitating the distribution of LNP-based treatments. Moreover, our lyophilized LNPs also presented unaltered thermo-stability at room temperature for 4 weeks. Considering that, until now, mRNA vaccines have been strictly stored at below-zero temperatures, this offers a great opportunity to lower logistic costs and widen the distribution of LNP-based therapeutics.

## 3. Materials and Methods

### 3.1. Materials

1-Octylnonyl 8-((2-hydroxyethyl) (6-oxo-6-(undecyloxy)hexyl)amino)octanoate (SM-102) ionizable lipid was purchase from BOC Sciences (NY, USA). The helper lipid 1,2-dioleoyl-sn-glycero-3-phosphoethanolamine (DOPE) was obtained from Corden Pharma (Boulder, CO, USA), cholesterol was purchased from Merck (Darmstadt, Germany), and 1,2-dimyristoyl-rac-glycero-3-methoxypolyethylene glycol-2000 (DMG-PEG2000) was obtained from Cayman Chemical (Ann Arbor, MI, USA). Citrate buffer was purchased from Thermo Scientific (Waltham, MA, USA). Ethanol, maltose, sucrose, Tris (tris(hydroxymethyl)aminomethane), PBS (phosphate-buffered saline) buffers and other chemicals were obtained from PanReac AppliChem ITW Reagents (Barcelona, Spain).

### 3.2. mRNA Production

mRNA was produced by in vitro transcription (IVT) as previously reported [28]. Prior to RNA IVT, pDNA encoding firefly luciferase (pTLuc) and green fluorescent protein (pTGFP) was linearized using the BspQI (NEB) enzyme, according to the manufacturer’s instructions. The linearization reaction was then purified with the Wizard^®^SV Gel and PCR Clean-Up (Promega, Madison, WI, USA), in accordance with the manufacturer’s instructions.

The purified linear DNA was subsequently employed for mRNA production by in vitro transcription using T7 RNA polymerase, following the manufacturer’s instructions. Briefly, transcription reactions were performed at 37 °C for 3 h using the following materials: template linear DNA (50 μg/mL); T7 RNA polymerase (5000 U/mL; HONGENE, Shanghai, China); RNase inhibitor (1000 U/mL, HONGENE, ON-039); inorganic pyrophosphatase (2 U/mL, HONGENE, ON-025); ATP (5 μg/mL, HONGENE, R1331); GTP (5 μg/mL, HONGENE, R2331); CTP (5 μg/mL, HONGENE, R3331); N1-Methylpseudouridine (5 μg/mL, HONGENE, R5-027); CleanCap^®^ AG (4 μg/mL, TRILINK^®^, San Diego, CA, USA); RNAse-free double-distilled water.

### 3.3. mRNA Purification

The generated mRNA transcripts were initially purified using DNaseI incubation (NEB, Ipswich, MA, USA) according to the manufacturer’s instructions. Following that, additional purification was carried out through affinity chromatography using POROS Oligo (dT) 25 columns (ThermoFisher, Waltham, MA, USA). Specifically, the buffers we employed were as follows: Buffer A, which contained 50 mM disodium phosphate, 0.5 M NaCl, and 5 mM EDTA, with pH = 7.0; and Buffer B, which contained 50 mM sodium dihydrogen phosphate and 5 mM EDTA, with pH = 7.0. The mRNA samples were initially half-diluted in Buffer A 2×. Following this, the column was equilibrated with 100% Buffer A, loaded with mRNA, washed with Buffer B, and ultimately eluted using double-deionized water. To completely eliminate Buffer B, mRNA was washed with a 30 KDa Amicon filter and then equilibrated through a one-tenth dilution in citrate buffer 10× with a pH of 6.5. The concentration of mRNA was determined by measuring the optical density at 260 nm, then adjusted to a final concentration of 1 mg/mL, aliquoted, and stored at −80 °C until needed. For quality assurance, all mRNAs underwent analysis through automated capillary electrophoresis (2100 Bioanalyzer G2938B, Agilent, Santa Clara, CA, USA). Subsequently, the mRNA samples were aliquoted and stored at −80 °C until needed.

### 3.4. Lipid Nanoparticle (LNP) Formulation

The microfluidic method was used for the controlled synthesis of LNPs. An ethanolic solution containing an ionizable lipid (SM-102), a helper lipid DOPE, cholesterol, and DMG-PEG2000 at a molar ratio of 50:10:38.5:1.5 was combined with an aqueous phase containing LUC-encoding mRNA diluted in 10 mM citrate buffer at pH 4, to achieve an ionizable lipid/RNA weight ratio of 10:1. A NanoAssemblr^®^ Ignite microfluidic device (Precision Nanosystems, Vancouver, Canada) was set to operate at a total flow rate of 12 mL/min and a aqueous–ethanol volume ratio of 3. After this, ethanol was removed by dialysis (Pur-A-Lyzer™ Midi Dialysis Kit, Sigma, Sant Louis, MO, USA) using Tris or PBS and the different lyoprotectants for the buffer exchange. The resulting formulation was adjusted to 100 μg/mL mRNA concentration and lyophilized and/or stored as required for subsequent stability studies.

### 3.5. Lyophilization Process

Lyophilization was performed in a Virtis Genesis Pilot Freeze Dryer (SP, Warminster, PA, USA). The lyophilization process is based on three stages: firstly, samples undergo a freezing step, followed by a primary drying step and a subsequent secondary drying step. The experimental conditions of the initial lyophilization protocol and the modified protocol are depicted in Tables S1 and S2, respectively. Vials were backfilled with pure nitrogen, capped, and transferred to various temperatures for stability assessments. To reconstitute lyophilized samples, 300 μL of RNase-free water was added to each vial and softly mixed until the solution turned into a homogeneous slightly white clear suspension.

### 3.6. LNP Characterization

LNP formulations were characterized to obtain the physicochemical characteristic parameters critical to their biological performance, such as particle size, polydispersity, and payload encapsulation. The average size, polydispersity (PDI), and zeta potential of LNPs were determined using a Malvern Zetasizer Advance Lab Blue Label (Malvern Instruments Ltd., Worcestershire, UK) by using a capillary cell (DTS1070) and diluting the sample (typically 1:100) in a filtered solution of 10 mM KCl.

The concentration and encapsulation efficiency of mRNA in LNPs were measured using a Quant-iT™ RiboGreen™ RNA Assay Kit from Thermo Fisher Scientific (Waltham, MA, USA), following the manufacturer’s protocols. Thus, the % of RNA encapsulated was calculated by comparing the total RNA obtained by the lysis of mRNA-LNPs using 0.5% Triton X-100 and the non-encapsulated RNA obtained when the LNPs are not lysed in the absence of detergent. Fluorescence was quantified in a Fluostar Omega microplate reader (BMG Labtech, Ortenberg, Germany). The EE% was calculated by the following equation:

$$Encapsulation\;Efficiency\;\%\left(EE\%\right)=\frac{RNA\;encapsulated\;in\;nanoparticles}{Total\;amount\;of\;RNA}\times 100$$

Agarose gel electrophoresis was additionally used to determine the encapsulation of mRNA in LNPs. The quantification of encapsulated mRNA was determined by band densitometry using ImageJ software. Briefly, pixel densities of the upper, slow-migrating bands (corresponding to LNP-encapsulated mRNA) and of the lower, fast-migrating bands (corresponding to free mRNA) were measured separately, and the percentage of encapsulated mRNA was calculated by dividing the amount of encapsulated mRNA by the total amount of mRNA, corresponding to the sum of the upper and the lower bands within the same lane. Samples were loaded in a 1% agarose gel including SYBR-Safe, and electrophoresis was run at 120 V for 30 min. Gels were visualized with a UV transilluminator iBright™ CL750 imaging system (Thermo Fisher Scientific, Waltham, MA, USA), using adequate exposure times to avoid image saturation.

mRNA integrity was determined by capillary electrophoresis on the Agilent 2100 Bioanalyzer (Agilent Technologies, Palo Alto, CA, USA) using the Agilent RNA 6000 Nano Kit (Agilent, Santa Clara, CA, USA) [29]. For the analysis of mRNA integrity, LNPs were disrupted by the addition of 0.5% Triton X-100 followed by heating at 70 °C during 2 min. As a reference of RNA integrity, the non-encapsulated RNA stock was treated and measured under the same conditions as the LNP samples, and the % of integrity was calculated relative to the integrity of this non-encapsulated RNA. Electropherograms obtained at each time point were analyzed using RNA 2100 Expert Software (version B.02.10).

### 3.7. Cryo-TEM Imaging of Fresh and Lyophilized LNPs

All CryoTEM analyses, including sample preparation and image collection, were performed using the CIC-bioGUNE Electron Microscopy Platform (Bilbao, Spain). For preparing the samples, L-polylysine was deposited in freshly glow-discharged carbon-only grids (EMResolutions, Newcastle, UK)), and then the grids were placed inside the chamber of the EM GP2 Automatic Plunge Freezing device (Leica, Wetzlar, Germany), which was maintained at 8 °C temperature and relative humidity close to saturation (90% RH). Four microliters of the sample was dropped onto the grid for 30 s. After incubation, most of the liquid on the grid was removed by blotting with absorbent standard filter paper (Ø55 mm, Grade 595, Hahnemühle, Dassel, Germany). After the blotting step, the grid was abruptly plunged into a liquid ethane bath, automatically set to −184 °C. Once the specimen was frozen, the vitrified grid was removed from the plunger and stored under liquid nitrogen inside a cryo-grid storage box.

The cryo-TEM data collection of the samples was performed on a JEM-1230 (JEOL Europe, Croissy, France) transmission electron microscope operated at 100 kV. This microscope has an UltraScan 4000 SP (4096 × 4096 pixels) cooled slow-scan CCD camera (GATAN, Leicester, UK). The images were recorded using DigitalMicrograph™ (Gatan Inc., Leicester, UK, https://www.gatan.com/products/tem-analysis/gatan-microscopy-suite-software) software at nominal magnifications of 10.000× and 25.000× with a pixel size of 1.106 nm and 0.473 nm, respectively. TEM images were analyzed using the open-source image processing software ImageJ (version 1.52i) to obtain the size distributions of LNPs.

### 3.8. Cell Culture and mRNA Transfection

HeLa (ACC57, DSMZ GmbH, Berlin, Germany) cells were cultured on high-glucose DMEM (Merck D6429, Darmstadt, Germany) supplemented with fetal bovine serum 10% (Sigma F7524, Sant Louis, MO, USA), penicillin–streptomycin solution 1% (GibcoTM, 15140122, Waltham, MA, USA), and Glutamax 2 mM (Fisher 35050038, Waltham, MA, USA). HEK-293T (ATCC HB-8065) cells were cultured on RPMI 1640 (GibcoTM, 31870074, Waltham, MA, USA) supplemented with fetal bovine serum 10% (Sigma F7524, Sant Louis, MO, USA), penicillin–streptomycin solution 1% (GibcoTM, 15140122, Waltham, MA, USA), and Glutamax 2 mM (Fisher 35050038, Waltham, MA, USA). Both cell lines were cultured in a 175 m^2^ flask. The day before transfection, cells were detached from the flask by trypsinization (11590626, Fisher, Waltham, MA, USA), and subsequently, they were seeded into 96-well plates at a density of 1 × 10^4^ cells/well. For transfection with a commercial cationic lipid, culture media was replaced with 90 μL of fresh media. Subsequently, a mixture of each mRNA (100 ng/well) and Lipofectamine MessengerMAXTM (Invitrogen 15397974; 0.2 μL/well, Waltham, MA, USA) was pre-incubated in OptiMEM media (31985062, Fisher, Waltham, MA, USA). The mRNA–lipofectamine mixture was added to the corresponding well in triplicate, directly resulting in a final mRNA concentration of 100 ng/well. Alternatively, the mRNA–lipofectamine mixture was diluted to half or a quarter of its concentration and then added to the cell culture, achieving final mRNA concentrations of 50 ng/well and 25 ng/well, respectively. For transfection with mRNA-LNPs, serial one-half dilutions in culture media were initially prepared. Then, 25 μL/well of the corresponding mRNA-LNP was added in triplicates to 100 μL of cells culture, resulting in final mRNA concentrations of 100 ng/well, 50 ng/well, or 25 ng/well. The cells, along with the mRNA-LNPs, were incubated for 24 h at 37 °C in a 5% CO_2_ atmosphere.

### 3.9. Firefly Luciferase Activity Quantification In Vitro

Cells were lysed 24 h post transfection by adding 100 μL of PBS-Triton 0.1%. Then, 98 μL of cell lysate was transferred to an opaque 96-well white plate. Buffered d-Luciferin (GoldBio LUCK−100 (St. Louis MO, USA) in 100 mM Tris-HCl pH 7.8, 5 mM MgCl_2_, 250 μM CoA, 150 μM ATP buffer) was added in 102 μL to each well, reaching a final concentration of 150 μg/mL. Cells that had not been incubated with any mRNA were employed as the negative control. Luminescence was measured after 5 min of incubation at room temperature in a FLUOstar Omega plate reader (BMG LABTECH, Ortenberg, Germany).

### 3.10. Statistical Analysis

Computer-based statistical analysis was carried out using the Prism^®^ software (version 10.3.1, GraphPad Software, San Diego, CA, USA). All values are expressed as mean ± standard deviation (SD) of at least 3 experiments. Statistical significance was analyzed by using Student’s *t*-test. A *p* < 0.05 was considered statistically significant.

### 3.11. In Vivo Activity in Mice

Female BALB/c mice (Charles River Laboratories, Wilmington, MA, USA), 8–10-weeks old and weighting 18–23 g, were acclimatized to new conditions upon arrival at the experimental facilities for 3–7 days. Housing conditions were maintained at a room temperature of 20–24 °C, humidity of 50–70%, and light intensity of 60 lux, with a light–dark cycle of 12 h. For the measurement of firefly luciferase activity in mice, LNPs produced as described above, containing 1 μg of the indicated mRNA in a final volume of 50 μL, were injected intramuscularly. At 4 and 24 h post mRNA-LPN inoculation, mice were anesthetized by inhalation with 4% isoflurane using a vaporizer. The maintenance of anesthesia was sustained at 1.5% of isoflurane. Then, D-luciferin (12507, Quimigen, Madrid, Spain) was intraperitoneally injected at 150 mg/kg, typically 200 μL of the stock at 15 mg/mL in PBS for a 20 g mouse. Luciferase images were captured 10 min after luciferin inoculation using the IVIS Lumina XRMS Imaging System (PerkinElmer, Waltham, MA, USA) following the manufacturer’s instructions.

All procedures were carried out under Project Licence 59/21 approved by the Ethic Committee for Animal Experiments from the University of Zaragoza. The care and use of animals were performed accordingly with the Spanish Policy for Animal Protection RD53/2013, which meets the European Union Directive 2010/63 on the protection of animals used for experimental and other scientific purposes. Image acquisition was carried out by the “Imagen Médica y Fenotipado” service at Instituto Aragonés de Ciencias de la Salud.

## 4. Conclusions

In the present study, we carried out a comprehensive optimization of the freeze-drying process, from the selection of the best buffers and cryoprotectants to the fine-tuning of the lyophilization parameters, seeking to preserve the physicochemical properties and functionality of lyophilized LNPs. Our results revealed that Tris buffer was more effective than PBS in maintaining LNPs’ integrity and bioactivity, correlating with reduced RNA leakage and smaller size increments. On the other hand, the optimization of the freeze-drying recipe involved adjusting the temperatures of the primary and secondary drying steps and extending the primary drying phase to ensure complete water removal. These modifications led to improved mRNA encapsulation and particle size consistency, with sucrose and maltose used as lyoprotectants at 20% *w*/*v* yielding the best results. We next assessed the long-term stabilities of LNPs lyophilized using the optimized parameters and stored under various conditions over 12 months, including non-lyophilized formulations at 4 °C, −20 °C, and −80 °C and lyophilized LNPs stored at 4 °C, 25 °C, and 37 °C. The results demonstrated that lyophilized LNPs stored at 4 °C maintained their physicochemical properties, such as particle size, polydispersity index (PDI), zeta potential, and mRNA encapsulation efficiency, comparably to freshly prepared LNPs. Importantly, lyophilized LNPs stored at 4 °C retained in vivo functionality, achieving similar luciferase expression in mice to non-lyophilized LNPs stored at −80 °C. Conversely, LNPs stored at 25 °C and 37 °C showed decreased mRNA integrity and transfection efficiency over time, although, remarkably, some functionality persisted for up to 60 weeks at room temperature. In summary, herein we have described a detailed optimization process for obtaining lyophilized LNPs that are able to retain their physicochemical properties and in vivo activity for up to one year, offering significant advantages for the storage and distribution of mRNA vaccines by enabling long-term stability at 4 °C and potentially reducing cold chain logistics costs.

## Data Availability

All data presented in this study are available on request to the corresponding author.

## Supplementary Materials

The following supporting information can be downloaded at: https://www.mdpi.com/article/10.3390/ijms251910603/s1.
