# Supplementary material for: Comprehensive Optimization of a Freeze-Drying Process Achieving Enhanced Long-Term Stability and In Vivo Performance of Lyophilized mRNA-LNPs
Source: Int J Mol Sci. 2024 Oct 1;25(19):10603. doi: 10.3390/ijms251910603 (PMC11476828; doi:10.3390/ijms251910603)

Table S1. Main process parameters of the initial freeze-drying process

	Freezing	Primary drying	Secondary drying
Temperature (ºC)	-50	-10	40
Ramp rate (ºC/min)	1.6	0.44	0.14
Time (h)	3.5	5	14
Vacuum (mTorr)	-	180	120 and 50

Table S2. Main process parameters of the modified freeze-drying process

	Freezing	Primary drying	Secondary drying
Temperature (ºC)	-50	-15	30
Ramp rate (ºC/min)	1.6	0.5	0.5
Time (h)	5	12	7
Vacuum (mTorr)	-	180	100

Figure S1. Images of freeze-dried mRNA-LNPs obtained using the initial lyophilization protocol in presence of Tris or PBS buffers and sucrose or maltose as lyoprotectants.

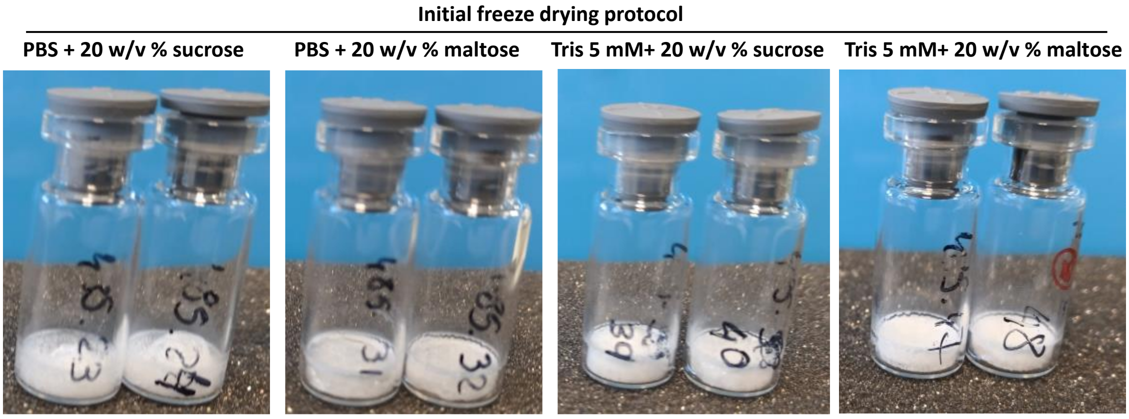

Figure S2. Curves of initial lyophilization recipe showing temperature and vacuum parameters during the lyophilization process.

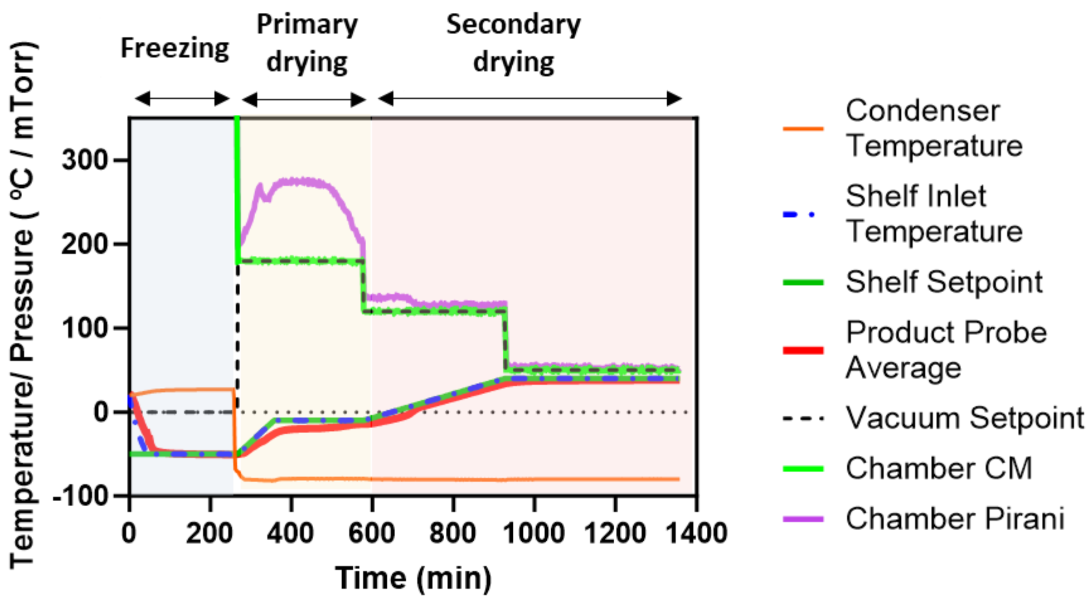

The difference in pressure between Pirani gauge and capacitance manometer reveals the presence of water during the primary drying. At the beginning of secondary drying the Pirani pressure has not yet converged to the capacitance, so the end of primary drying is not reach.

Figure S3. Figure S3. Curves of optimized lyophilization recipe showing temperature and vacuum parameters during the lyophilization process.

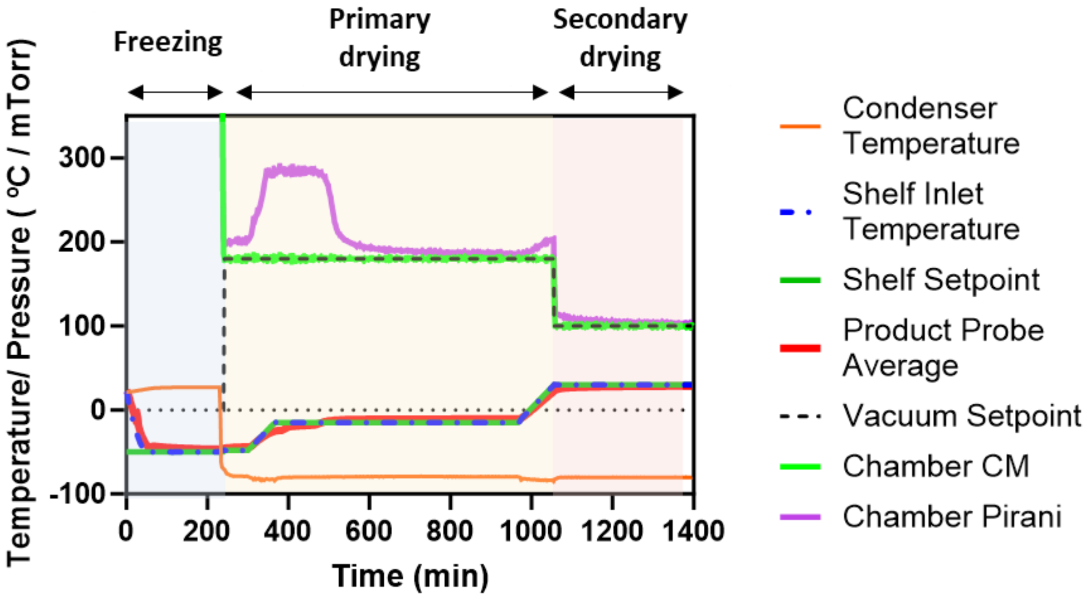

Figure S4. Physicochemical characterization of freshly prepared and freeze-dried LNPs prepared using the optimized method.

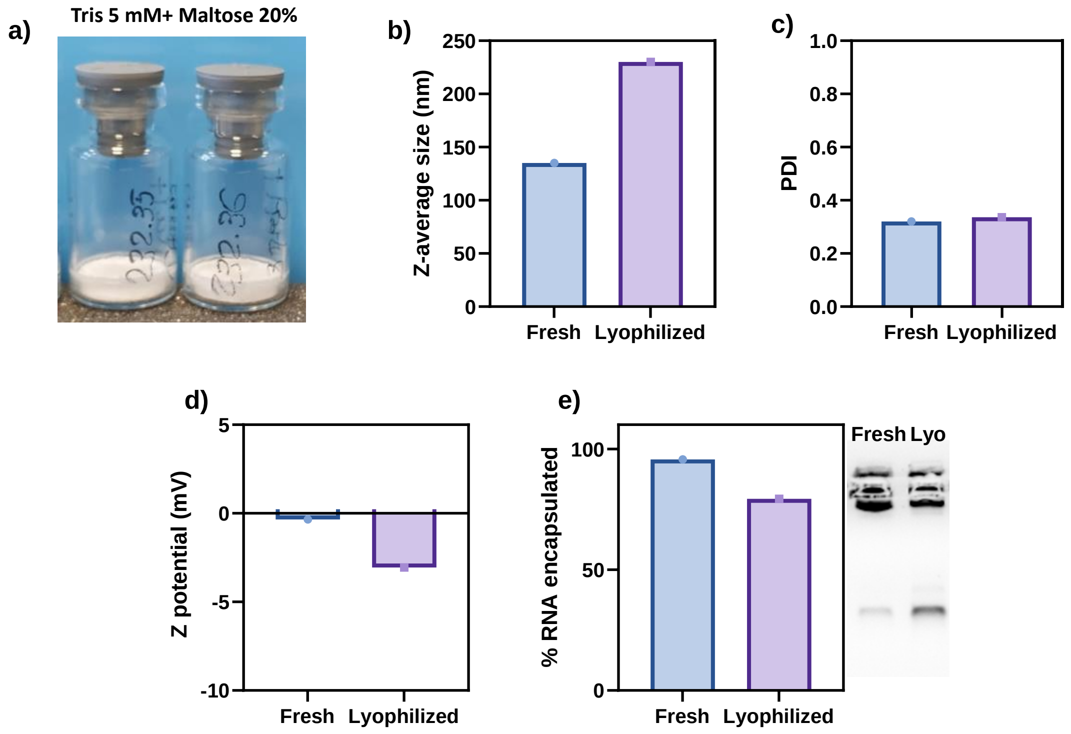

(a) Image of freeze-dried mRNA-LNPs obtained using the optimized lyophilization protocol in presence of maltose as lyoprotectant. (b) Particle size (C) polydispersity (D) Z potential (e) encapsulation efficiency of mRNA (%) obtained by densitometry from the image of agarose gel electrophoresis.

Figure S5. Stability of fresh (4ºC) and frozen LNPs stored in Tris 5 mM containing 15% w/v sucrose at -20ºC and -80ºC for 32 weeks.

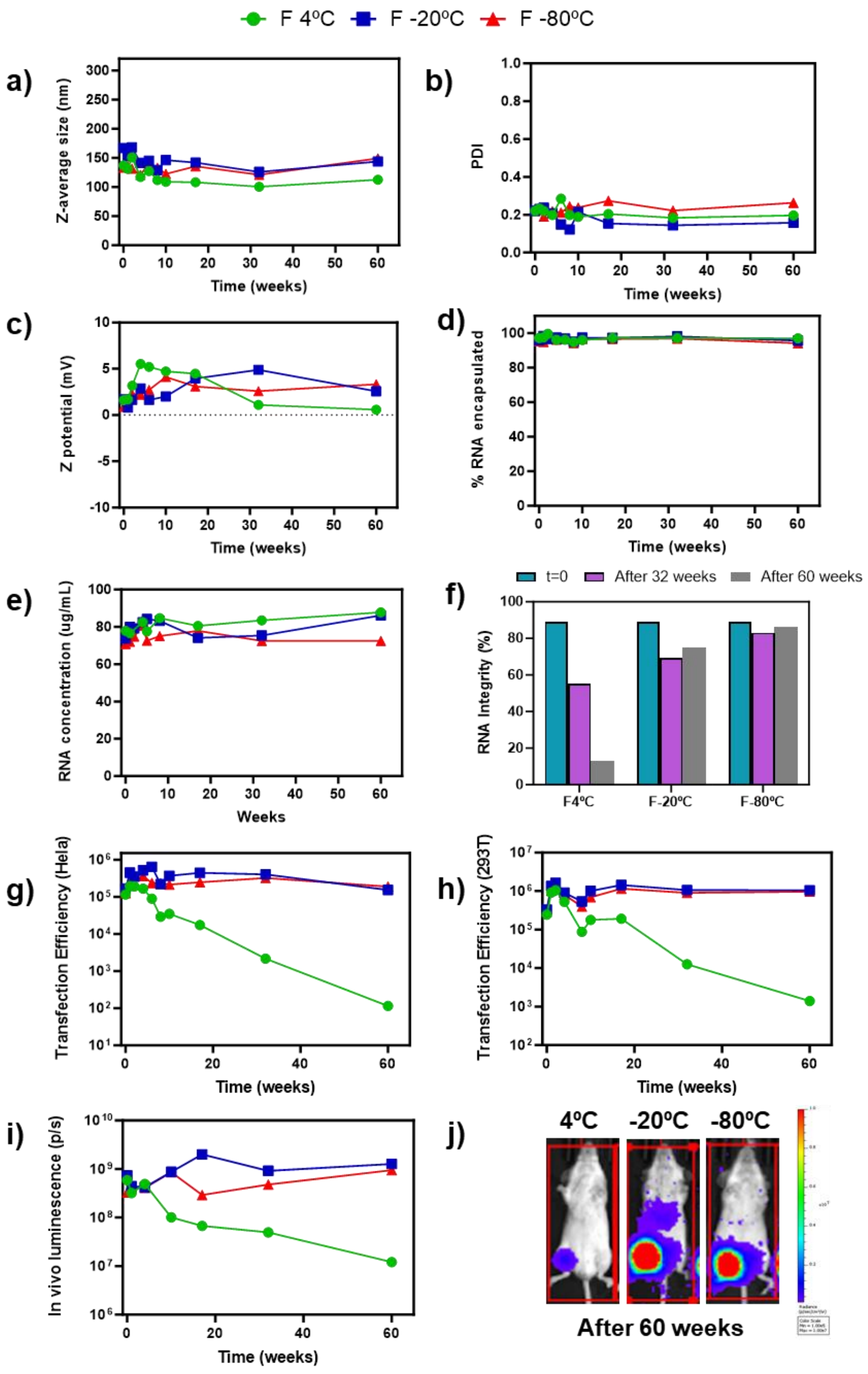

(a) Particle size (b) polydispersity (c) Z potential (d) encapsulation efficiency of mRNA (%) (e) mRNA concentration obtained by RiboGreen assay and (f) mRNA integrity (%) obtained by capillary electrophoresis. (g-h) Transfection efficiency of LNPs in (g) HeLa and (h) 293T cell lines. (i) Average luminescence radiance and (j) luminescence images of mice intramuscularly treated with 1µg of mRNA-LNPs stored at 4ºC, -20 or -80 ºC for 32 weeks.

Figure S6. Cryo-TEM images of liquid LNPs freshly prepared and after 60 weeks stored at -80ºC.

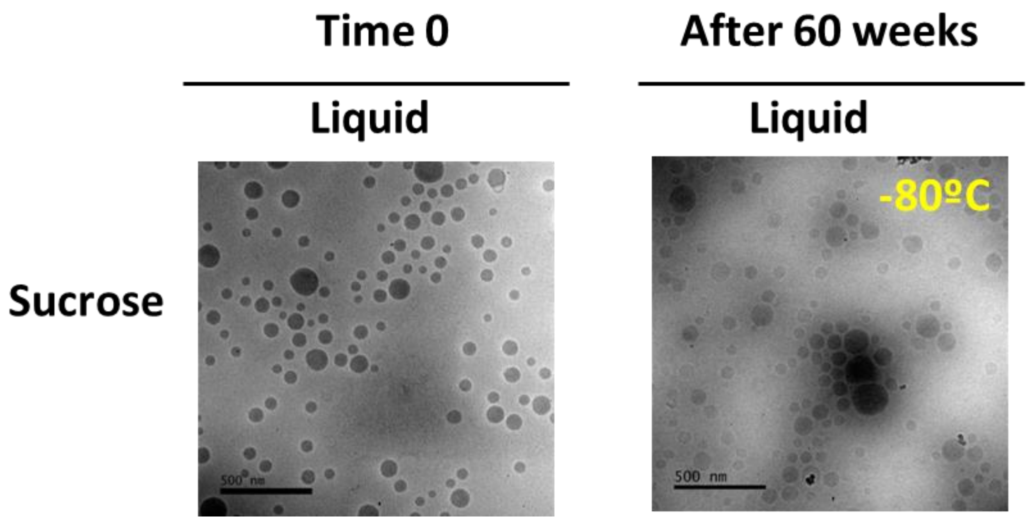

Figure S7. Schematic summary of the workflow followed

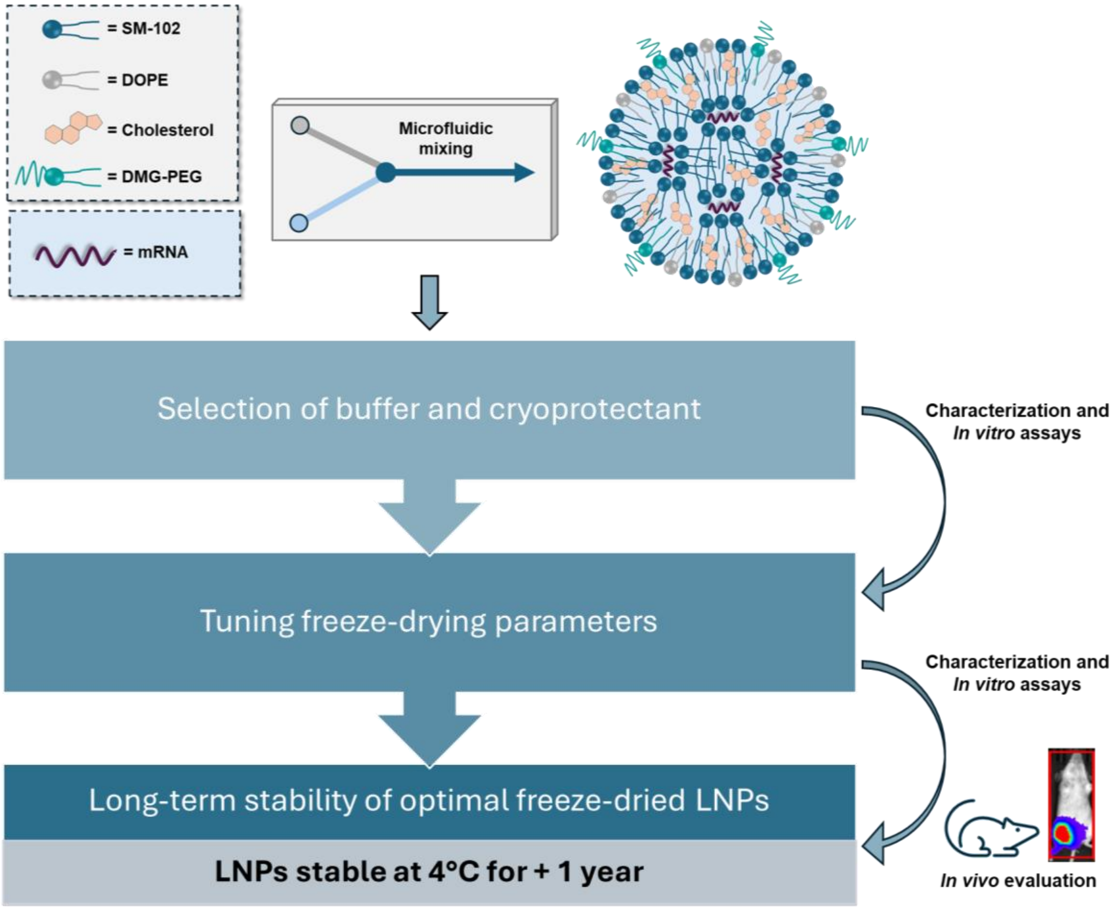

Supplement: Supplementary file 1 [file ijms-25-10603-s001.zip › ijms-3196125-supplementary.pdf]
